# [5,11,17,23-Tetra-*tert*-butyl-25,27-(3,6-dioxaoctan-1,8-di­oxy)-26,28-bis­(pyridin-2-ylmeth­oxy)calix[4]arene]sodium iodide–1,2,4,5-tetra­fluoro-3,6-diiodo­benzene–methanol (2/3/4)

**DOI:** 10.1107/S1600536813007757

**Published:** 2013-04-05

**Authors:** Gabriella Cavallo, Pierangelo Metrangolo, Melchiorre F. Parisi, Tullio Pilati, Giuseppe Resnati, Giancarlo Terraneo

**Affiliations:** a NFMLab, Department of Chemistry, Materials and Chemical Engineering, "G. Natta", Politecnico di Milano, Via Mancinelli, 7, I-20131 Milano, Italy; b Dipartimento di Chimica Organica e Biologica, Universitá di Messina, Salita Sperone 31, I-98166 Messina, Italy

## Abstract

The title compound, [Na(C_62_H_76_N_2_O_6_)]I·1.5C_6_F_4_I_2_·2CH_3_OH, is composed of five components: a calix[4]arene derivative (hereinafter C4), a sodium cation, an iodide anion, a 1,2,4,5-tetra­fluoro-3,6-diiodo­benzene (tFdIB) mol­ecule and a methanol mol­ecule in a 1:1:1:1.5:2 ratio. The complex shows several inter­esting features: (i) the polyoxygenated loop of C4 effectively chelates a sodium cation in the form of a distorted octahedron and separates it from the iodide counter-ion, the shortest Na^+^⋯I^−^ distance being greater than 6.5 Å; (ii) the cavity of C4 is filled by a methanol mol­ecule; (iii) a second methanol mol­ecule is hydrogen-bonded to the N atom of a pyridinyl substituent pendant of C4 and halogen-bonded to the I atom of a tFdIB mol­ecule; (iv) the two I atoms of another tFdIB mol­ecule are halogen-bonded to two iodide anions, which act as monodentate halogen-bond acceptorss; (v) one of the two tFdIB molecules is located about a centre of inversion.

## Related literature

For applications of calix[4]arenes derivative, see: Dondoni & Marra (2010 ▶). When calix-crown-arenes coordinate potassium (Gattuso *et al.*, 2006 ▶) or caesium cations (Gattuso *et al.*, 2007 ▶), the resulting naked iodide anions form XBs with diiodo­perfluoro­alkanes. Alternatively, the N atoms of pyridyl pendants at the lower rim of calixarenes form halogen bonds with tetra­fluoro-diiodo­benzene (Messina *et al.*, 2000 ▶). For a description of the Cambridge Structural Database, see: Allen (2002 ▶).
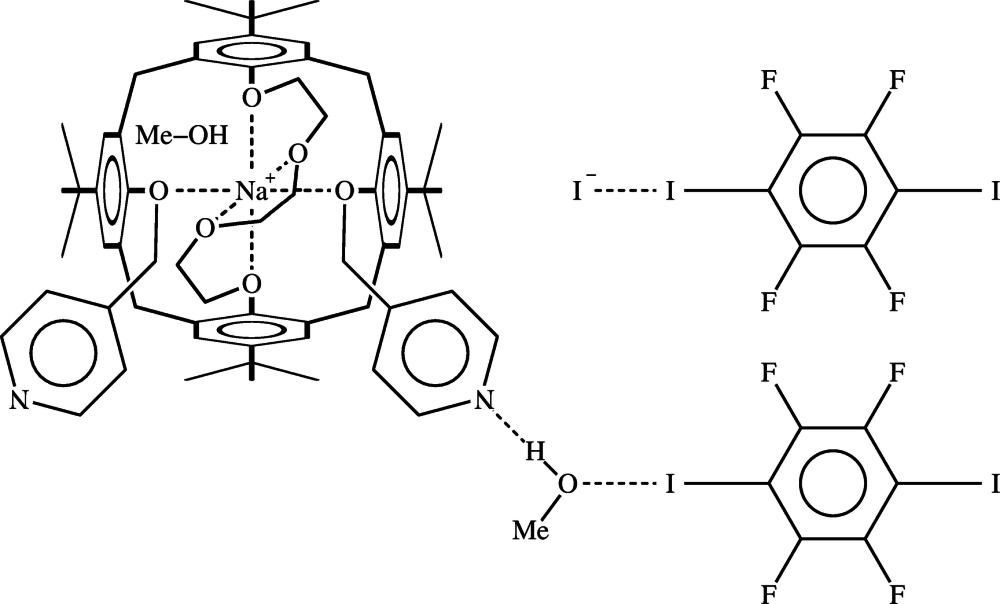


## Experimental

### 

#### Crystal data

[Na(C_62_H_76_N_2_O_6_)]I·1.5C_6_F_4_I_2_·2CH_4_O
*M* *_r_* = 1762.01
Triclinic, 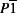

*a* = 13.4019 (12) Å
*b* = 14.2255 (12) Å
*c* = 21.022 (2) Å
α = 85.90 (2)°
β = 84.61 (2)°
γ = 65.75 (2)°
*V* = 3635.6 (8) Å^3^
*Z* = 2
Mo *K*α radiation
μ = 1.79 mm^−1^
*T* = 90 K
0.42 × 0.30 × 0.20 mm

#### Data collection

Bruker APEXII CCD diffractometer
Absorption correction: multi-scan (*SADABS*; Bruker, 2004 ▶) *T*_min_ = 0.866, *T*_max_ = 1.000
88833 measured reflections
33353 independent reflections
29534 reflections with *I* > 2σ(*I*)
*R*_int_ = 0.029

#### Refinement

*R*[*F* ^2^ > 2σ(*F* ^2^)] = 0.045
*wR*(*F* ^2^) = 0.095
*S* = 1.13
33353 reflections
847 parameters
H-atom parameters constrained
Δρ_max_ = 2.33 e Å^−3^
Δρ_min_ = −1.52 e Å^−3^

### 

Data collection: *APEX2* (Bruker, 2004 ▶); cell refinement: *SAINT* (Bruker, 2004 ▶); data reduction: *SAINT*; program(s) used to solve structure: *SIR2002* (Burla *et al.*, 2003 ▶); program(s) used to refine structure: *SHELXL2012* (Sheldrick, 2008 ▶); molecular graphics: *ORTEP-3 for Windows* (Farrugia, 2012 ▶) and *Mercury* (Macrae *et al.*, 2006 ▶); software used to prepare material for publication: *SHELXL2012*.

## Supplementary Material

Crystal structure: contains datablock(s) global, I. DOI: 10.1107/S1600536813007757/bg2501sup1.cif

Structure factors: contains datablock(s) I. DOI: 10.1107/S1600536813007757/bg2501Isup2.hkl

Additional supplementary materials:  crystallographic information; 3D view; checkCIF report

## Figures and Tables

**Table 1:** Halogen and hydrogen-bond geometry (Å, °)

*Z*—*X*⋯*Y*	*Z*—*X*	*X*⋯*Y*	*Z*⋯*Y*	*Z*—*X*⋯*Y*
C63—I1⋯O7		2.75 (1)		179 (1)
C69—I3⋯I4		3.38 (1)		175 (1)
C66—I2⋯F6^i^		3.21 (1)		149 (1)
O7—H7*O*7⋯N2	0.84	2.00	2.809 (3)	161

Symmetry code: (i) 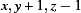.

**Fig. 1.**
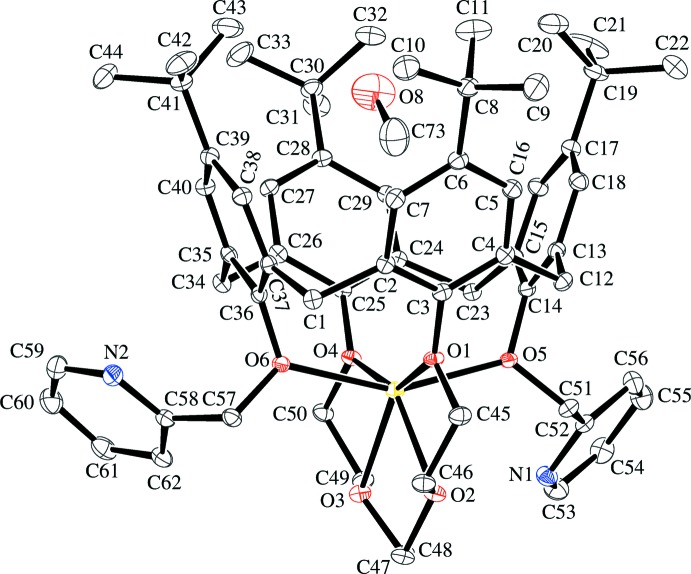
The ORTEP-3 view of C4Na+ supramolecular cation with the methanol molecule filling the cavity. Ellipsoids at 50% level. H atoms and the remaining components of the structure are omitted for clarity.

**Fig. 2.**
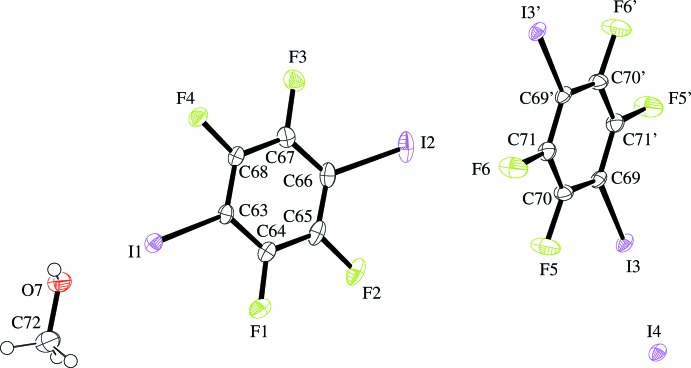
: The ORTEP-3 view of the components not reported in Fig. 1, namely I-···tFdIB···I- and tFdIB···MeOH. The orientation adopted for these components is different from that adopted in Fig. 1.

**Fig. 3.**
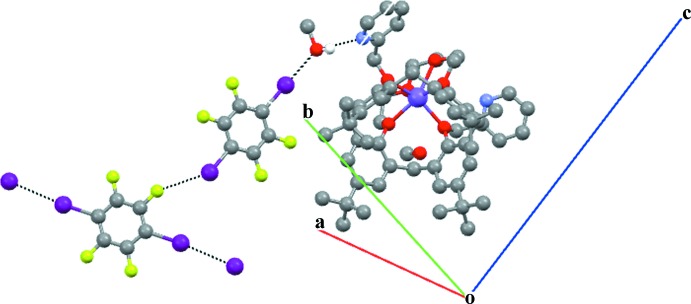
Mercury ball and stick view of two asymmetric units around the symmetry center. Short XBs and HB are reported. Hydrogen atoms not involved in H-bonding are omitted for clarity.

## supplementary crystallographic information

### Comment

Calix[4]arenes derivatives are very popular compounds in host/guest supramolecular chemistry and find applications in diverse fields spanning from non linear optics to catalysis (Dondoni & Marra, 2010). Calix-crown-arenes, namely calixarenes with a polyoxyethylene chain bridging two positions at the lower rim, are frequently used to chelate metal cations. In several structures, crown loops formed by five or six glycol units effectively coordinate large metal cations. Only nine calix-crown-arenes contain smaller loops formed by four glycol groups. They are CAWYEF, CAZCUB, CAZDUC, which have two free phenolic functions on the lower rim, and GUDMAU, GUDMEY, GUDMIC, XEGSAE, XEGSEI, XEGSIM, which have two propyloxy functions (code from CSD-Version 5.33 Nov 2011; Allen, 2002). We have already reported that when calix-crown-arenes coordinate potassium (Gattuso, *et al.*, 2006) or caesium cations (Gattuso, *et al.*, 2007), the resulting naked iodide anions form XBs with diiodoperfluoroalkanes. Alternatively, the nitrogen atoms of pyridyl pendants at the lower rim of calixarenes form XBs with tetrafluoro-diiodobenzene (tFdIB) (Messina, *et al.*, 2000). This paper presents a calix-crown-arene having, at the lower rim, two 2-pyridylmethoxy groups and a short loop (compounded by four glycol units connecting positions 25 and 27) which cryptates the small Na^+^ cation from NaI. The resulting supramolecular salt might act as a polydentate XB donor towards tFdIB, as both pyridyl nitrogen atoms and naked iodide anions can function as donors of electron density (XB acceptors) to iodoperfluorocarbons when singly present in structurally similar systems. Usually, iodide anions are more effective XB acceptors than lone pair possessing nitrogen atoms. In the stucture described here, pyridine nitrogen atoms form no XB. One nitrogen atom is engaged in an intramolecular contact with a hydrogen atom of the glycol loop and the other is linked to a methanol molecule (O7···N2 2.809 (3) Å) *via* a quite short hydrogen bond (HB). Another methanol molecule occupies the calixarene cavity. It shows no disorder and the hydroxyl H atom and a methyl H atom are weakly linked to the inner faces of two adjacent aromatic rings. The two iodine atoms of one tFdIB molecule are halogen bonded to two iodide anions which work as monodentate XB acceptors. The coordination sphere of iodide anions is completed by three H···I^-^ contacts with three supramolecular cations surrounding the anion. One iodine atom of the other independent tFdIB molecule is engaged in a quite short XB with the oxygen of a MeOH molecule and the other iodine atom forms a quite long contact with a fluorine atom of another tFdIB molecule. Figure 1 shows the supramolecular cation with the included methanol molecule. Figure 2 shows the other components of the system with the numbering scheme. Figure 3 is a view of the main HBs and XBs of the structure.

### Experimental

Methanol solutions of C4, sodium iodide, and tFdIB were prepared separately and mixed in a single vial which was closed in a wide mouth flask containing vaseline oil. The solvent was allowed to diffuse at room temperature until good quality crystals were formed.

### Refinement

Hydrogen atoms were positioned geometrically and refined using a riding model, with C—H = 0.95–0.99 Å and with *U*_iso_(H) = 1.2 (1.5 for methyl groups and hydroxyl group) times *U*_eq_(C).

### Crystal data

**Table:**

[Na(C_62_H_76_N_2_O_6_)]I·1.5C_6_F_4_I_2_·2CH_4_O	*Z* = 2
*M**_r_* = 1762.01	*F*(000) = 1754
Triclinic, *P*1	*D*_x_ = 1.610 Mg m^−^^3^
Hall symbol: -P 1	Mo *K*α radiation, λ = 0.71073 Å
*a* = 13.4019 (12) Å	Cell parameters from 33174 reflections
*b* = 14.2255 (12) Å	θ = 2.5–36.2°
*c* = 21.022 (2) Å	µ = 1.79 mm^−^^1^
α = 85.90 (2)°	*T* = 90 K
β = 84.61 (2)°	Prism, colourless
γ = 65.75 (2)°	0.42 × 0.30 × 0.20 mm
*V* = 3635.6 (8) Å^3^	

### Data collection

**Table:**

Bruker APEXII CCD diffractometer	29534 reflections with *I* > 2σ(*I*)
Radiation source: sealed tube	*R*_int_ = 0.029
φ and ω scans	θ_max_ = 36.4°, θ_min_ = 1.7°
Absorption correction: multi-scan (*SADABS*; Bruker, 2004)	*h* = −21→22
*T*_min_ = 0.866, *T*_max_ = 1.000	*k* = −23→23
88833 measured reflections	*l* = −34→35
33353 independent reflections	

### Refinement

**Table:**

Refinement on *F*^2^	Primary atom site location: structure-invariant direct methods
Least-squares matrix: full	Secondary atom site location: difference Fourier map
*R*[*F*^2^ > 2σ(*F*^2^)] = 0.045	Hydrogen site location: inferred from neighbouring sites
*wR*(*F*^2^) = 0.095	H-atom parameters constrained
*S* = 1.13	*w* = 1/[σ^2^(*F*_o_^2^) + (0.0333*P*)^2^ + 3.1236*P*], where *P* = (*F*_o_^2^ + 2*F*_c_^2^)/3
33353 reflections	(Δ/σ)_max_ = 0.002
847 parameters	Δρ_max_ = 2.33 e Å^−^^3^
0 restraints	Δρ_min_ = −1.52 e Å^−^^3^

### Special details

**Table:**

Experimental. Bruker KRYOFLEX low temperature device.
Geometry. All e.s.d.'s (except the e.s.d. in the dihedral angle between two l.s. planes) are estimated using the full covariance matrix. The cell e.s.d.'s are taken into account individually in the estimation of e.s.d.'s in distances, angles and torsion angles; correlations between e.s.d.'s in cell parameters are only used when they are defined by crystal symmetry. An approximate (isotropic) treatment of cell e.s.d.'s is used for estimating e.s.d.'s involving l.s. planes.

### Fractional atomic coordinates and isotropic or equivalent isotropic displacement parameters (Å^2^)

**Table:**

	*x*	*y*	*z*	*U*_iso_*/*U*_eq_	
I1	0.35237 (2)	0.90822 (2)	0.08989 (2)	0.01904 (3)	
I2	0.34600 (2)	0.94345 (2)	−0.24227 (2)	0.03038 (4)	
F1	0.38098 (13)	1.07085 (11)	−0.01520 (7)	0.0281 (3)	
F2	0.37976 (14)	1.08362 (12)	−0.14181 (8)	0.0324 (3)	
F3	0.34131 (13)	0.76760 (12)	−0.13909 (7)	0.0274 (3)	
F4	0.34360 (12)	0.75421 (10)	−0.01259 (7)	0.0225 (3)	
C63	0.36335 (17)	0.91197 (16)	−0.00969 (10)	0.0169 (3)	
C64	0.37373 (18)	0.99386 (17)	−0.04493 (11)	0.0204 (4)	
C65	0.37311 (18)	1.00061 (18)	−0.11085 (11)	0.0228 (4)	
C66	0.36154 (18)	0.92572 (19)	−0.14444 (11)	0.0220 (4)	
C67	0.35349 (18)	0.84232 (18)	−0.10952 (11)	0.0208 (4)	
C68	0.35475 (17)	0.83579 (16)	−0.04374 (10)	0.0175 (4)	
I3	0.66213 (2)	0.13414 (2)	0.44197 (2)	0.01774 (3)	
F5	0.53244 (16)	0.12749 (14)	0.57729 (8)	0.0365 (4)	
F6	0.40813 (15)	0.02901 (14)	0.62120 (7)	0.0352 (4)	
C69	0.56585 (16)	0.05310 (15)	0.47630 (10)	0.0157 (3)	
C70	0.51796 (19)	0.06462 (17)	0.53805 (10)	0.0191 (4)	
C71	0.45316 (18)	0.01311 (17)	0.56077 (10)	0.0189 (4)	
I4	0.81611 (2)	0.26919 (2)	0.40053 (2)	0.01718 (3)	
Na1	0.28660 (6)	0.48248 (6)	0.33541 (4)	0.01198 (14)	
C1	0.35809 (15)	0.61807 (14)	0.16785 (9)	0.0123 (3)	
H1A	0.3913	0.6537	0.1362	0.015*	
H1B	0.3985	0.6022	0.2070	0.015*	
C2	0.36498 (15)	0.51884 (14)	0.14083 (9)	0.0122 (3)	
C3	0.37844 (15)	0.43217 (15)	0.18038 (9)	0.0118 (3)	
C4	0.36586 (15)	0.34727 (15)	0.15885 (9)	0.0125 (3)	
C5	0.34566 (16)	0.34878 (15)	0.09473 (9)	0.0138 (3)	
H5	0.3378	0.2912	0.0793	0.017*	
C6	0.33659 (16)	0.43189 (15)	0.05242 (9)	0.0138 (3)	
C7	0.34621 (16)	0.51596 (15)	0.07682 (9)	0.0139 (3)	
H7	0.3398	0.5735	0.0490	0.017*	
C8	0.31192 (17)	0.43014 (16)	−0.01729 (9)	0.0170 (4)	
C9	0.3974 (2)	0.3326 (2)	−0.04814 (11)	0.0287 (5)	
H9A	0.4706	0.3320	−0.0471	0.043*	
H9B	0.3814	0.3318	−0.0926	0.043*	
H9C	0.3948	0.2716	−0.0245	0.043*	
C10	0.3157 (2)	0.5231 (2)	−0.05771 (11)	0.0275 (5)	
H10A	0.2616	0.5870	−0.0392	0.041*	
H10B	0.2989	0.5191	−0.1015	0.041*	
H10C	0.3892	0.5225	−0.0581	0.041*	
C11	0.1975 (2)	0.4313 (2)	−0.01892 (12)	0.0314 (6)	
H11A	0.1428	0.4939	0.0008	0.047*	
H11B	0.1948	0.3705	0.0048	0.047*	
H11C	0.1815	0.4304	−0.0634	0.047*	
C12	0.36723 (15)	0.25868 (15)	0.20404 (9)	0.0126 (3)	
H12A	0.4120	0.2535	0.2401	0.015*	
H12B	0.4021	0.1933	0.1811	0.015*	
C13	0.25217 (15)	0.27271 (14)	0.23036 (9)	0.0116 (3)	
C14	0.20958 (15)	0.30877 (14)	0.29168 (9)	0.0108 (3)	
C15	0.10034 (15)	0.33158 (14)	0.31206 (9)	0.0109 (3)	
C16	0.03657 (16)	0.30926 (15)	0.27155 (9)	0.0136 (3)	
H16	−0.0373	0.3224	0.2855	0.016*	
C17	0.07715 (16)	0.26863 (15)	0.21161 (9)	0.0143 (3)	
C18	0.18510 (16)	0.25254 (15)	0.19188 (9)	0.0140 (3)	
H18	0.2138	0.2268	0.1506	0.017*	
C19	0.00734 (17)	0.24537 (17)	0.16685 (10)	0.0182 (4)	
C20	−0.0057 (3)	0.3158 (2)	0.10669 (13)	0.0333 (6)	
H20A	0.0669	0.3052	0.0865	0.050*	
H20B	−0.0452	0.3879	0.1184	0.050*	
H20C	−0.0472	0.2991	0.0766	0.050*	
C21	0.0657 (2)	0.1323 (2)	0.14800 (14)	0.0323 (6)	
H21A	0.1391	0.1197	0.1282	0.048*	
H21B	0.0232	0.1179	0.1175	0.048*	
H21C	0.0722	0.0871	0.1862	0.048*	
C22	−0.1062 (2)	0.2629 (3)	0.19732 (15)	0.0424 (8)	
H22A	−0.1475	0.2470	0.1668	0.064*	
H22B	−0.1450	0.3351	0.2091	0.064*	
H22C	−0.0990	0.2179	0.2357	0.064*	
C23	0.04498 (15)	0.38614 (14)	0.37376 (9)	0.0110 (3)	
H23A	−0.0005	0.3522	0.3962	0.013*	
H23B	0.1012	0.3824	0.4024	0.013*	
C24	−0.02688 (15)	0.49852 (14)	0.35701 (8)	0.0106 (3)	
C25	0.01333 (14)	0.57485 (14)	0.35639 (8)	0.0104 (3)	
C26	−0.04157 (15)	0.67315 (14)	0.32869 (9)	0.0108 (3)	
C27	−0.14493 (15)	0.69689 (15)	0.30689 (9)	0.0122 (3)	
H27	−0.1840	0.7636	0.2884	0.015*	
C28	−0.19249 (15)	0.62560 (15)	0.31141 (9)	0.0120 (3)	
C29	−0.13076 (15)	0.52614 (14)	0.33562 (9)	0.0116 (3)	
H29	−0.1607	0.4759	0.3375	0.014*	
C30	−0.30827 (16)	0.65194 (16)	0.29161 (10)	0.0160 (3)	
C31	−0.37864 (17)	0.64144 (19)	0.35165 (11)	0.0211 (4)	
H31A	−0.4533	0.6578	0.3402	0.032*	
H31B	−0.3808	0.6893	0.3836	0.032*	
H31C	−0.3466	0.5706	0.3694	0.032*	
C32	−0.30418 (19)	0.5761 (2)	0.24192 (11)	0.0226 (4)	
H32A	−0.3785	0.5932	0.2295	0.034*	
H32B	−0.2739	0.5057	0.2604	0.034*	
H32C	−0.2576	0.5812	0.2042	0.034*	
C33	−0.3618 (2)	0.7615 (2)	0.26342 (15)	0.0311 (6)	
H33A	−0.4354	0.7747	0.2515	0.047*	
H33B	−0.3172	0.7695	0.2254	0.047*	
H33C	−0.3670	0.8107	0.2952	0.047*	
C34	0.01059 (15)	0.75009 (14)	0.32028 (9)	0.0117 (3)	
H34A	0.0626	0.7360	0.3538	0.014*	
H34B	−0.0471	0.8206	0.3259	0.014*	
C35	0.07130 (15)	0.74521 (14)	0.25480 (9)	0.0108 (3)	
C36	0.18597 (15)	0.69558 (14)	0.24474 (9)	0.0107 (3)	
C37	0.23745 (15)	0.68700 (14)	0.18334 (9)	0.0114 (3)	
C38	0.17334 (15)	0.73731 (15)	0.13246 (9)	0.0127 (3)	
H38	0.2084	0.7353	0.0908	0.015*	
C39	0.05995 (15)	0.79027 (15)	0.14072 (9)	0.0123 (3)	
C40	0.01065 (15)	0.79118 (15)	0.20240 (9)	0.0124 (3)	
H40	−0.0670	0.8244	0.2087	0.015*	
C41	−0.01023 (16)	0.84392 (16)	0.08496 (9)	0.0154 (3)	
C42	0.0597 (2)	0.8482 (2)	0.02385 (11)	0.0257 (5)	
H42A	0.0117	0.8830	−0.0107	0.039*	
H42B	0.1085	0.7779	0.0117	0.039*	
H42C	0.1037	0.8864	0.0314	0.039*	
C43	−0.0810 (2)	0.7861 (2)	0.07241 (13)	0.0300 (5)	
H43A	−0.1262	0.8203	0.0366	0.045*	
H43B	−0.1286	0.7862	0.1108	0.045*	
H43C	−0.0333	0.7147	0.0618	0.045*	
C44	−0.08552 (19)	0.95498 (18)	0.10161 (11)	0.0234 (4)	
H44A	−0.1305	0.9892	0.0658	0.035*	
H44B	−0.0410	0.9922	0.1099	0.035*	
H44C	−0.1333	0.9546	0.1398	0.035*	
O1	0.40231 (11)	0.43285 (11)	0.24340 (6)	0.0120 (2)	
C45	0.51836 (15)	0.38562 (16)	0.25212 (10)	0.0154 (3)	
H45A	0.5595	0.4022	0.2146	0.018*	
H45B	0.5438	0.3097	0.2565	0.018*	
C46	0.53821 (16)	0.42634 (17)	0.31143 (10)	0.0158 (3)	
H46A	0.6164	0.3907	0.3206	0.019*	
H46B	0.5199	0.5011	0.3054	0.019*	
O2	0.47052 (12)	0.40849 (12)	0.36326 (7)	0.0147 (3)	
C47	0.48480 (16)	0.44350 (16)	0.42234 (9)	0.0152 (3)	
H47A	0.4825	0.5140	0.4163	0.018*	
H47B	0.5566	0.3972	0.4383	0.018*	
C48	0.39328 (16)	0.44326 (16)	0.46926 (9)	0.0150 (3)	
H48A	0.3939	0.3733	0.4741	0.018*	
H48B	0.4018	0.4643	0.5116	0.018*	
O3	0.29249 (11)	0.51493 (11)	0.44475 (7)	0.0136 (2)	
C49	0.19918 (16)	0.51223 (16)	0.48210 (9)	0.0139 (3)	
H49A	0.1959	0.5368	0.5255	0.017*	
H49B	0.2039	0.4408	0.4862	0.017*	
C50	0.09859 (16)	0.58125 (15)	0.44879 (9)	0.0134 (3)	
H50A	0.0327	0.5752	0.4708	0.016*	
H50B	0.0888	0.6540	0.4497	0.016*	
O4	0.11237 (11)	0.55024 (11)	0.38350 (6)	0.0114 (2)	
O5	0.27846 (11)	0.32323 (10)	0.33249 (6)	0.0114 (2)	
C51	0.34873 (15)	0.22820 (15)	0.36477 (9)	0.0132 (3)	
H51A	0.4129	0.2364	0.3791	0.016*	
H51B	0.3759	0.1712	0.3346	0.016*	
C52	0.28679 (15)	0.20185 (14)	0.42148 (9)	0.0123 (3)	
N1	0.28355 (15)	0.24742 (14)	0.47572 (8)	0.0157 (3)	
C53	0.22346 (19)	0.23120 (18)	0.52636 (10)	0.0195 (4)	
H53	0.2209	0.2629	0.5651	0.023*	
C54	0.16501 (18)	0.17105 (17)	0.52556 (10)	0.0201 (4)	
H54	0.1226	0.1626	0.5626	0.024*	
C55	0.16956 (19)	0.12328 (17)	0.46959 (11)	0.0204 (4)	
H55	0.1309	0.0807	0.4676	0.024*	
C56	0.23156 (18)	0.13859 (16)	0.41647 (10)	0.0174 (4)	
H56	0.2363	0.1066	0.3775	0.021*	
O6	0.24875 (11)	0.65174 (10)	0.29724 (6)	0.0115 (2)	
C57	0.30091 (16)	0.71421 (15)	0.31892 (9)	0.0137 (3)	
H57A	0.3406	0.7342	0.2820	0.016*	
H57B	0.3550	0.6732	0.3500	0.016*	
C58	0.21752 (16)	0.80947 (15)	0.34979 (9)	0.0132 (3)	
N2	0.17300 (16)	0.89317 (14)	0.31092 (9)	0.0179 (3)	
C59	0.0882 (2)	0.97461 (18)	0.33517 (12)	0.0238 (4)	
H59	0.0564	1.0342	0.3081	0.029*	
C60	0.0444 (2)	0.97661 (19)	0.39779 (12)	0.0254 (5)	
H60	−0.0187	1.0344	0.4125	0.031*	
C61	0.09456 (19)	0.89269 (18)	0.43829 (11)	0.0214 (4)	
H61	0.0690	0.8932	0.4820	0.026*	
C62	0.18275 (17)	0.80765 (16)	0.41415 (10)	0.0161 (3)	
H62	0.2189	0.7491	0.4411	0.019*	
O7	0.33656 (17)	0.90617 (14)	0.22139 (8)	0.0283 (4)	
H7O7	0.2800	0.9027	0.2403	0.042*	
C72	0.3633 (2)	0.9777 (2)	0.25185 (14)	0.0315 (5)	
H72A	0.4250	0.9866	0.2275	0.047*	
H72B	0.3837	0.9517	0.2953	0.047*	
H72C	0.2997	1.0442	0.2539	0.047*	
O8	−0.0537 (2)	0.5639 (2)	0.16897 (13)	0.0536 (7)	
H8O8	−0.0759	0.5807	0.2069	0.080*	
C73	0.0623 (3)	0.5265 (2)	0.16222 (17)	0.0409 (7)	
H73A	0.0838	0.5847	0.1606	0.061*	
H73B	0.0897	0.4888	0.1226	0.061*	
H73C	0.0934	0.4802	0.1988	0.061*	

### Atomic displacement parameters (Å^2^)

**Table:**

	*U*^11^	*U*^22^	*U*^33^	*U*^12^	*U*^13^	*U*^23^
I1	0.02211 (6)	0.01769 (6)	0.01537 (6)	−0.00697 (5)	0.00277 (5)	−0.00068 (4)
I2	0.02485 (7)	0.03401 (9)	0.01512 (6)	0.00398 (6)	0.00076 (5)	0.00473 (6)
F1	0.0363 (8)	0.0219 (7)	0.0295 (8)	−0.0157 (6)	−0.0029 (6)	0.0030 (6)
F2	0.0371 (9)	0.0287 (8)	0.0293 (8)	−0.0140 (7)	−0.0020 (6)	0.0148 (6)
F3	0.0313 (8)	0.0266 (7)	0.0217 (7)	−0.0084 (6)	−0.0027 (6)	−0.0052 (6)
F4	0.0296 (7)	0.0183 (6)	0.0205 (6)	−0.0115 (5)	−0.0003 (5)	0.0025 (5)
C63	0.0153 (8)	0.0174 (9)	0.0153 (8)	−0.0048 (7)	0.0006 (7)	0.0020 (7)
C64	0.0191 (9)	0.0172 (9)	0.0222 (10)	−0.0054 (7)	−0.0006 (8)	0.0025 (7)
C65	0.0182 (9)	0.0212 (10)	0.0232 (10)	−0.0045 (8)	0.0017 (8)	0.0090 (8)
C66	0.0159 (9)	0.0258 (11)	0.0170 (9)	−0.0021 (8)	−0.0001 (7)	0.0045 (8)
C67	0.0185 (9)	0.0217 (10)	0.0182 (9)	−0.0043 (8)	−0.0006 (7)	−0.0016 (8)
C68	0.0156 (8)	0.0161 (8)	0.0175 (9)	−0.0039 (7)	0.0004 (7)	0.0025 (7)
I3	0.01577 (6)	0.01619 (6)	0.02253 (6)	−0.00874 (5)	−0.00138 (5)	0.00536 (5)
F5	0.0587 (11)	0.0454 (10)	0.0245 (7)	−0.0411 (9)	0.0089 (7)	−0.0126 (7)
F6	0.0538 (10)	0.0465 (10)	0.0195 (7)	−0.0370 (9)	0.0149 (7)	−0.0106 (7)
C69	0.0139 (8)	0.0144 (8)	0.0197 (9)	−0.0074 (7)	−0.0008 (7)	0.0041 (7)
C70	0.0247 (10)	0.0197 (9)	0.0176 (9)	−0.0140 (8)	0.0007 (7)	−0.0013 (7)
C71	0.0219 (9)	0.0201 (9)	0.0158 (8)	−0.0106 (8)	0.0022 (7)	0.0008 (7)
I4	0.01597 (6)	0.01691 (6)	0.02085 (6)	−0.00969 (5)	−0.00064 (4)	0.00334 (4)
Na1	0.0119 (3)	0.0131 (3)	0.0112 (3)	−0.0053 (3)	−0.0009 (3)	−0.0002 (3)
C1	0.0111 (7)	0.0137 (7)	0.0124 (7)	−0.0059 (6)	0.0016 (6)	−0.0006 (6)
C2	0.0127 (7)	0.0129 (7)	0.0100 (7)	−0.0044 (6)	0.0013 (6)	−0.0006 (6)
C3	0.0105 (7)	0.0145 (8)	0.0088 (7)	−0.0038 (6)	0.0007 (6)	−0.0007 (6)
C4	0.0113 (7)	0.0132 (7)	0.0118 (7)	−0.0041 (6)	0.0012 (6)	−0.0010 (6)
C5	0.0153 (8)	0.0148 (8)	0.0106 (7)	−0.0054 (6)	0.0004 (6)	−0.0020 (6)
C6	0.0137 (8)	0.0157 (8)	0.0097 (7)	−0.0037 (6)	0.0003 (6)	−0.0019 (6)
C7	0.0138 (8)	0.0139 (8)	0.0118 (7)	−0.0039 (6)	0.0009 (6)	−0.0001 (6)
C8	0.0195 (9)	0.0182 (9)	0.0106 (7)	−0.0048 (7)	−0.0014 (7)	−0.0018 (6)
C9	0.0375 (13)	0.0244 (11)	0.0148 (9)	−0.0027 (10)	−0.0006 (9)	−0.0047 (8)
C10	0.0409 (14)	0.0264 (11)	0.0145 (9)	−0.0127 (10)	−0.0059 (9)	0.0038 (8)
C11	0.0267 (12)	0.0479 (16)	0.0210 (11)	−0.0161 (12)	−0.0075 (9)	0.0029 (11)
C12	0.0110 (7)	0.0127 (7)	0.0120 (7)	−0.0028 (6)	−0.0001 (6)	0.0000 (6)
C13	0.0117 (7)	0.0115 (7)	0.0103 (7)	−0.0037 (6)	−0.0007 (6)	0.0001 (6)
C14	0.0111 (7)	0.0111 (7)	0.0106 (7)	−0.0047 (6)	−0.0023 (6)	0.0006 (6)
C15	0.0110 (7)	0.0122 (7)	0.0102 (7)	−0.0055 (6)	−0.0012 (6)	0.0002 (6)
C16	0.0123 (7)	0.0157 (8)	0.0141 (8)	−0.0067 (6)	−0.0010 (6)	−0.0016 (6)
C17	0.0160 (8)	0.0142 (8)	0.0134 (8)	−0.0063 (7)	−0.0030 (6)	−0.0010 (6)
C18	0.0150 (8)	0.0147 (8)	0.0122 (7)	−0.0057 (6)	−0.0007 (6)	−0.0019 (6)
C19	0.0180 (9)	0.0238 (10)	0.0156 (8)	−0.0101 (8)	−0.0044 (7)	−0.0054 (7)
C20	0.0423 (15)	0.0349 (14)	0.0288 (12)	−0.0189 (12)	−0.0202 (11)	0.0044 (10)
C21	0.0397 (14)	0.0249 (12)	0.0365 (14)	−0.0139 (11)	−0.0157 (12)	−0.0060 (10)
C22	0.0258 (12)	0.078 (2)	0.0356 (15)	−0.0310 (15)	0.0007 (11)	−0.0240 (15)
C23	0.0102 (7)	0.0124 (7)	0.0103 (7)	−0.0047 (6)	−0.0004 (6)	0.0001 (6)
C24	0.0104 (7)	0.0119 (7)	0.0096 (7)	−0.0049 (6)	0.0004 (5)	−0.0007 (6)
C25	0.0086 (7)	0.0140 (7)	0.0093 (7)	−0.0053 (6)	0.0003 (5)	−0.0013 (6)
C26	0.0104 (7)	0.0129 (7)	0.0105 (7)	−0.0064 (6)	0.0020 (6)	−0.0018 (6)
C27	0.0115 (7)	0.0128 (7)	0.0117 (7)	−0.0046 (6)	−0.0013 (6)	0.0017 (6)
C28	0.0105 (7)	0.0150 (8)	0.0113 (7)	−0.0062 (6)	−0.0008 (6)	0.0010 (6)
C29	0.0121 (7)	0.0137 (7)	0.0106 (7)	−0.0068 (6)	−0.0001 (6)	−0.0004 (6)
C30	0.0117 (7)	0.0172 (8)	0.0204 (9)	−0.0070 (7)	−0.0049 (7)	0.0031 (7)
C31	0.0134 (8)	0.0279 (11)	0.0234 (10)	−0.0099 (8)	0.0022 (7)	−0.0046 (8)
C32	0.0197 (9)	0.0343 (12)	0.0183 (9)	−0.0150 (9)	−0.0048 (8)	−0.0005 (8)
C33	0.0206 (10)	0.0239 (11)	0.0517 (16)	−0.0111 (9)	−0.0197 (11)	0.0154 (11)
C34	0.0127 (7)	0.0127 (7)	0.0110 (7)	−0.0066 (6)	0.0011 (6)	−0.0018 (6)
C35	0.0125 (7)	0.0111 (7)	0.0109 (7)	−0.0071 (6)	0.0005 (6)	−0.0012 (6)
C36	0.0123 (7)	0.0097 (7)	0.0104 (7)	−0.0048 (6)	−0.0005 (6)	0.0001 (5)
C37	0.0113 (7)	0.0106 (7)	0.0128 (7)	−0.0052 (6)	0.0008 (6)	−0.0014 (6)
C38	0.0129 (7)	0.0146 (8)	0.0097 (7)	−0.0051 (6)	0.0007 (6)	−0.0012 (6)
C39	0.0131 (7)	0.0131 (7)	0.0109 (7)	−0.0058 (6)	−0.0006 (6)	0.0005 (6)
C40	0.0115 (7)	0.0141 (8)	0.0119 (7)	−0.0057 (6)	−0.0006 (6)	0.0002 (6)
C41	0.0155 (8)	0.0180 (8)	0.0125 (8)	−0.0066 (7)	−0.0026 (6)	0.0020 (6)
C42	0.0225 (10)	0.0323 (12)	0.0137 (9)	−0.0034 (9)	−0.0014 (8)	0.0053 (8)
C43	0.0349 (13)	0.0360 (14)	0.0283 (12)	−0.0219 (11)	−0.0165 (10)	0.0057 (10)
C44	0.0221 (10)	0.0197 (10)	0.0205 (10)	−0.0007 (8)	−0.0032 (8)	0.0034 (8)
O1	0.0100 (5)	0.0155 (6)	0.0091 (5)	−0.0038 (5)	−0.0007 (4)	−0.0006 (5)
C45	0.0106 (7)	0.0192 (9)	0.0146 (8)	−0.0040 (7)	−0.0007 (6)	−0.0021 (7)
C46	0.0132 (8)	0.0206 (9)	0.0152 (8)	−0.0082 (7)	−0.0020 (6)	−0.0004 (7)
O2	0.0153 (6)	0.0197 (7)	0.0117 (6)	−0.0096 (5)	−0.0017 (5)	−0.0014 (5)
C47	0.0142 (8)	0.0209 (9)	0.0132 (8)	−0.0088 (7)	−0.0041 (6)	−0.0023 (7)
C48	0.0140 (8)	0.0192 (9)	0.0122 (7)	−0.0067 (7)	−0.0050 (6)	0.0011 (6)
O3	0.0121 (6)	0.0170 (6)	0.0121 (6)	−0.0061 (5)	−0.0018 (5)	0.0010 (5)
C49	0.0139 (8)	0.0169 (8)	0.0105 (7)	−0.0062 (7)	−0.0008 (6)	0.0008 (6)
C50	0.0133 (7)	0.0164 (8)	0.0100 (7)	−0.0053 (6)	0.0006 (6)	−0.0040 (6)
O4	0.0104 (5)	0.0150 (6)	0.0095 (5)	−0.0056 (5)	−0.0006 (4)	−0.0022 (4)
O5	0.0123 (6)	0.0118 (6)	0.0109 (5)	−0.0054 (5)	−0.0038 (4)	0.0015 (4)
C51	0.0119 (7)	0.0139 (8)	0.0125 (7)	−0.0040 (6)	−0.0021 (6)	0.0026 (6)
C52	0.0114 (7)	0.0121 (7)	0.0120 (7)	−0.0035 (6)	−0.0018 (6)	0.0018 (6)
N1	0.0190 (8)	0.0172 (7)	0.0123 (7)	−0.0088 (6)	−0.0008 (6)	−0.0012 (6)
C53	0.0230 (10)	0.0237 (10)	0.0125 (8)	−0.0104 (8)	0.0012 (7)	−0.0022 (7)
C54	0.0211 (9)	0.0209 (9)	0.0164 (9)	−0.0083 (8)	0.0038 (7)	0.0014 (7)
C55	0.0220 (10)	0.0194 (9)	0.0223 (10)	−0.0118 (8)	0.0015 (8)	0.0002 (8)
C56	0.0210 (9)	0.0157 (8)	0.0166 (8)	−0.0088 (7)	0.0006 (7)	−0.0018 (7)
O6	0.0129 (6)	0.0123 (6)	0.0107 (5)	−0.0065 (5)	−0.0026 (4)	0.0006 (4)
C57	0.0124 (7)	0.0151 (8)	0.0151 (8)	−0.0067 (6)	−0.0017 (6)	−0.0022 (6)
C58	0.0146 (8)	0.0149 (8)	0.0126 (7)	−0.0080 (6)	−0.0019 (6)	−0.0014 (6)
N2	0.0223 (8)	0.0154 (7)	0.0160 (7)	−0.0075 (7)	−0.0037 (6)	0.0005 (6)
C59	0.0291 (11)	0.0156 (9)	0.0228 (10)	−0.0039 (8)	−0.0069 (9)	−0.0015 (8)
C60	0.0236 (10)	0.0210 (10)	0.0276 (11)	−0.0034 (8)	−0.0019 (9)	−0.0107 (9)
C61	0.0227 (10)	0.0255 (10)	0.0175 (9)	−0.0108 (8)	0.0014 (8)	−0.0089 (8)
C62	0.0200 (9)	0.0178 (9)	0.0132 (8)	−0.0103 (7)	−0.0015 (7)	−0.0021 (7)
O7	0.0409 (10)	0.0284 (9)	0.0201 (8)	−0.0203 (8)	0.0069 (7)	−0.0034 (7)
C72	0.0371 (14)	0.0260 (12)	0.0330 (13)	−0.0151 (11)	0.0021 (11)	−0.0025 (10)
O8	0.0406 (13)	0.0715 (18)	0.0456 (14)	−0.0197 (13)	0.0048 (11)	−0.0123 (13)
C73	0.0376 (16)	0.0337 (15)	0.0500 (19)	−0.0146 (13)	0.0064 (13)	−0.0053 (13)

### Geometric parameters (Å, º)

**Table:**

I1—C63	2.083 (2)	C30—C31	1.540 (3)
I2—C66	2.074 (2)	C31—H31A	0.9800
F1—C64	1.340 (3)	C31—H31B	0.9800
F2—C65	1.339 (3)	C31—H31C	0.9800
F3—C67	1.341 (3)	C32—H32A	0.9800
F4—C68	1.347 (2)	C32—H32B	0.9800
C63—C64	1.384 (3)	C32—H32C	0.9800
C63—C68	1.389 (3)	C33—H33A	0.9800
C64—C65	1.383 (3)	C33—H33B	0.9800
C65—C66	1.386 (4)	C33—H33C	0.9800
C66—C67	1.387 (3)	C34—C35	1.523 (3)
C67—C68	1.381 (3)	C34—H34A	0.9900
I3—C69	2.1078 (19)	C34—H34B	0.9900
F5—C70	1.340 (3)	C35—C40	1.389 (3)
F6—C71	1.347 (3)	C35—C36	1.405 (3)
C69—C71^i^	1.378 (3)	C36—C37	1.394 (3)
C69—C70	1.382 (3)	C36—O6	1.400 (2)
C70—C71	1.383 (3)	C37—C38	1.397 (3)
C71—C69^i^	1.378 (3)	C38—C39	1.391 (3)
Na1—O4	2.2915 (16)	C38—H38	0.9500
Na1—O5	2.3178 (16)	C39—C40	1.397 (3)
Na1—O1	2.3188 (16)	C39—C41	1.527 (3)
Na1—O6	2.3443 (16)	C40—H40	0.9500
Na1—O2	2.3617 (17)	C41—C44	1.530 (3)
Na1—O3	2.3913 (16)	C41—C42	1.530 (3)
C1—C2	1.524 (3)	C41—C43	1.538 (3)
C1—C37	1.525 (3)	C42—H42A	0.9800
C1—H1A	0.9900	C42—H42B	0.9800
C1—H1B	0.9900	C42—H42C	0.9800
C2—C3	1.394 (3)	C43—H43A	0.9800
C2—C7	1.398 (3)	C43—H43B	0.9800
C3—O1	1.393 (2)	C43—H43C	0.9800
C3—C4	1.397 (3)	C44—H44A	0.9800
C4—C5	1.396 (3)	C44—H44B	0.9800
C4—C12	1.518 (3)	C44—H44C	0.9800
C5—C6	1.398 (3)	O1—C45	1.443 (2)
C5—H5	0.9500	C45—C46	1.501 (3)
C6—C7	1.392 (3)	C45—H45A	0.9900
C6—C8	1.536 (3)	C45—H45B	0.9900
C7—H7	0.9500	C46—O2	1.428 (2)
C8—C9	1.530 (3)	C46—H46A	0.9900
C8—C11	1.531 (3)	C46—H46B	0.9900
C8—C10	1.536 (3)	O2—C47	1.428 (2)
C9—H9A	0.9800	C47—C48	1.501 (3)
C9—H9B	0.9800	C47—H47A	0.9900
C9—H9C	0.9800	C47—H47B	0.9900
C10—H10A	0.9800	C48—O3	1.433 (2)
C10—H10B	0.9800	C48—H48A	0.9900
C10—H10C	0.9800	C48—H48B	0.9900
C11—H11A	0.9800	O3—C49	1.426 (2)
C11—H11B	0.9800	C49—C50	1.504 (3)
C11—H11C	0.9800	C49—H49A	0.9900
C12—C13	1.525 (3)	C49—H49B	0.9900
C12—H12A	0.9900	C50—O4	1.444 (2)
C12—H12B	0.9900	C50—H50A	0.9900
C13—C18	1.388 (3)	C50—H50B	0.9900
C13—C14	1.402 (3)	O5—C51	1.455 (2)
C14—C15	1.394 (3)	C51—C52	1.502 (3)
C14—O5	1.400 (2)	C51—H51A	0.9900
C15—C16	1.398 (3)	C51—H51B	0.9900
C15—C23	1.525 (3)	C52—N1	1.340 (3)
C16—C17	1.391 (3)	C52—C56	1.394 (3)
C16—H16	0.9500	N1—C53	1.338 (3)
C17—C18	1.395 (3)	C53—C54	1.379 (3)
C17—C19	1.528 (3)	C53—H53	0.9500
C18—H18	0.9500	C54—C55	1.385 (3)
C19—C22	1.521 (3)	C54—H54	0.9500
C19—C20	1.533 (4)	C55—C56	1.387 (3)
C19—C21	1.535 (3)	C55—H55	0.9500
C20—H20A	0.9800	C56—H56	0.9500
C20—H20B	0.9800	O6—C57	1.454 (2)
C20—H20C	0.9800	C57—C58	1.501 (3)
C21—H21A	0.9800	C57—H57A	0.9900
C21—H21B	0.9800	C57—H57B	0.9900
C21—H21C	0.9800	C58—N2	1.346 (3)
C22—H22A	0.9800	C58—C62	1.391 (3)
C22—H22B	0.9800	N2—C59	1.337 (3)
C22—H22C	0.9800	C59—C60	1.387 (4)
C23—C24	1.525 (3)	C59—H59	0.9500
C23—H23A	0.9900	C60—C61	1.380 (4)
C23—H23B	0.9900	C60—H60	0.9500
C24—C29	1.392 (3)	C61—C62	1.385 (3)
C24—C25	1.395 (3)	C61—H61	0.9500
C25—O4	1.393 (2)	C62—H62	0.9500
C25—C26	1.398 (3)	O7—C72	1.418 (3)
C26—C27	1.398 (3)	O7—H7O7	0.8400
C26—C34	1.515 (3)	C72—H72A	0.9800
C27—C28	1.396 (3)	C72—H72B	0.9800
C27—H27	0.9500	C72—H72C	0.9800
C28—C29	1.400 (3)	O8—C73	1.418 (4)
C28—C30	1.531 (3)	O8—H8O8	0.8400
C29—H29	0.9500	C73—H73A	0.9800
C30—C33	1.525 (3)	C73—H73B	0.9800
C30—C32	1.536 (3)	C73—H73C	0.9800
			
C64—C63—C68	116.9 (2)	C32—C30—C31	109.31 (17)
C64—C63—I1	121.17 (17)	C30—C31—H31A	109.5
C68—C63—I1	121.88 (15)	C30—C31—H31B	109.5
F1—C64—C65	118.3 (2)	H31A—C31—H31B	109.5
F1—C64—C63	120.1 (2)	C30—C31—H31C	109.5
C65—C64—C63	121.5 (2)	H31A—C31—H31C	109.5
F2—C65—C64	118.4 (2)	H31B—C31—H31C	109.5
F2—C65—C66	120.2 (2)	C30—C32—H32A	109.5
C64—C65—C66	121.3 (2)	C30—C32—H32B	109.5
C65—C66—C67	117.4 (2)	H32A—C32—H32B	109.5
C65—C66—I2	120.96 (17)	C30—C32—H32C	109.5
C67—C66—I2	121.48 (18)	H32A—C32—H32C	109.5
F3—C67—C68	118.7 (2)	H32B—C32—H32C	109.5
F3—C67—C66	120.4 (2)	C30—C33—H33A	109.5
C68—C67—C66	120.9 (2)	C30—C33—H33B	109.5
F4—C68—C67	117.9 (2)	H33A—C33—H33B	109.5
F4—C68—C63	120.14 (19)	C30—C33—H33C	109.5
C67—C68—C63	121.9 (2)	H33A—C33—H33C	109.5
C71^i^—C69—C70	116.57 (18)	H33B—C33—H33C	109.5
C71^i^—C69—I3	122.59 (15)	C26—C34—C35	112.19 (15)
C70—C69—I3	120.84 (16)	C26—C34—H34A	109.2
F5—C70—C69	120.37 (19)	C35—C34—H34A	109.2
F5—C70—C71	118.28 (19)	C26—C34—H34B	109.2
C69—C70—C71	121.3 (2)	C35—C34—H34B	109.2
F6—C71—C69^i^	119.86 (19)	H34A—C34—H34B	107.9
F6—C71—C70	118.1 (2)	C40—C35—C36	118.34 (17)
C69^i^—C71—C70	122.1 (2)	C40—C35—C34	118.62 (16)
O4—Na1—O5	90.28 (6)	C36—C35—C34	123.04 (16)
O4—Na1—O1	149.51 (6)	C37—C36—O6	119.79 (16)
O5—Na1—O1	85.04 (6)	C37—C36—C35	121.02 (17)
O4—Na1—O6	85.40 (6)	O6—C36—C35	119.17 (16)
O5—Na1—O6	153.33 (6)	C36—C37—C38	118.33 (17)
O1—Na1—O6	85.47 (6)	C36—C37—C1	123.48 (17)
O4—Na1—O2	139.63 (6)	C38—C37—C1	117.97 (16)
O5—Na1—O2	92.91 (6)	C39—C38—C37	122.22 (17)
O1—Na1—O2	70.80 (5)	C39—C38—H38	118.9
O6—Na1—O2	107.34 (6)	C37—C38—H38	118.9
O4—Na1—O3	70.07 (6)	C38—C39—C40	117.60 (17)
O5—Na1—O3	107.73 (6)	C38—C39—C41	122.19 (17)
O1—Na1—O3	139.84 (6)	C40—C39—C41	120.19 (17)
O6—Na1—O3	95.50 (6)	C35—C40—C39	122.24 (17)
O2—Na1—O3	70.66 (6)	C35—C40—H40	118.9
C2—C1—C37	108.24 (15)	C39—C40—H40	118.9
C2—C1—H1A	110.1	C39—C41—C44	109.63 (17)
C37—C1—H1A	110.1	C39—C41—C42	112.03 (17)
C2—C1—H1B	110.1	C44—C41—C42	107.59 (18)
C37—C1—H1B	110.1	C39—C41—C43	109.30 (17)
H1A—C1—H1B	108.4	C44—C41—C43	109.0 (2)
C3—C2—C7	118.03 (17)	C42—C41—C43	109.2 (2)
C3—C2—C1	121.10 (16)	C41—C42—H42A	109.5
C7—C2—C1	120.53 (17)	C41—C42—H42B	109.5
O1—C3—C2	118.19 (17)	H42A—C42—H42B	109.5
O1—C3—C4	120.02 (16)	C41—C42—H42C	109.5
C2—C3—C4	121.77 (17)	H42A—C42—H42C	109.5
C5—C4—C3	117.84 (17)	H42B—C42—H42C	109.5
C5—C4—C12	120.50 (17)	C41—C43—H43A	109.5
C3—C4—C12	121.56 (17)	C41—C43—H43B	109.5
C4—C5—C6	122.43 (18)	H43A—C43—H43B	109.5
C4—C5—H5	118.8	C41—C43—H43C	109.5
C6—C5—H5	118.8	H43A—C43—H43C	109.5
C7—C6—C5	117.40 (17)	H43B—C43—H43C	109.5
C7—C6—C8	122.46 (18)	C41—C44—H44A	109.5
C5—C6—C8	120.09 (18)	C41—C44—H44B	109.5
C6—C7—C2	122.37 (18)	H44A—C44—H44B	109.5
C6—C7—H7	118.8	C41—C44—H44C	109.5
C2—C7—H7	118.8	H44A—C44—H44C	109.5
C9—C8—C11	109.5 (2)	H44B—C44—H44C	109.5
C9—C8—C10	107.42 (19)	C3—O1—C45	113.05 (14)
C11—C8—C10	108.4 (2)	O1—C45—C46	108.41 (16)
C9—C8—C6	109.69 (17)	O1—C45—H45A	110.0
C11—C8—C6	109.39 (18)	C46—C45—H45A	110.0
C10—C8—C6	112.36 (18)	O1—C45—H45B	110.0
C8—C9—H9A	109.5	C46—C45—H45B	110.0
C8—C9—H9B	109.5	H45A—C45—H45B	108.4
H9A—C9—H9B	109.5	O2—C46—C45	108.21 (16)
C8—C9—H9C	109.5	O2—C46—H46A	110.1
H9A—C9—H9C	109.5	C45—C46—H46A	110.1
H9B—C9—H9C	109.5	O2—C46—H46B	110.1
C8—C10—H10A	109.5	C45—C46—H46B	110.1
C8—C10—H10B	109.5	H46A—C46—H46B	108.4
H10A—C10—H10B	109.5	C46—O2—C47	112.42 (15)
C8—C10—H10C	109.5	O2—C47—C48	107.57 (15)
H10A—C10—H10C	109.5	O2—C47—H47A	110.2
H10B—C10—H10C	109.5	C48—C47—H47A	110.2
C8—C11—H11A	109.5	O2—C47—H47B	110.2
C8—C11—H11B	109.5	C48—C47—H47B	110.2
H11A—C11—H11B	109.5	H47A—C47—H47B	108.5
C8—C11—H11C	109.5	O3—C48—C47	107.30 (16)
H11A—C11—H11C	109.5	O3—C48—H48A	110.3
H11B—C11—H11C	109.5	C47—C48—H48A	110.3
C4—C12—C13	112.14 (15)	O3—C48—H48B	110.3
C4—C12—H12A	109.2	C47—C48—H48B	110.3
C13—C12—H12A	109.2	H48A—C48—H48B	108.5
C4—C12—H12B	109.2	C49—O3—C48	111.92 (15)
C13—C12—H12B	109.2	O3—C49—C50	107.78 (15)
H12A—C12—H12B	107.9	O3—C49—H49A	110.2
C18—C13—C14	118.23 (17)	C50—C49—H49A	110.2
C18—C13—C12	119.32 (16)	O3—C49—H49B	110.2
C14—C13—C12	122.42 (16)	C50—C49—H49B	110.2
C15—C14—O5	119.88 (16)	H49A—C49—H49B	108.5
C15—C14—C13	121.28 (17)	O4—C50—C49	108.38 (15)
O5—C14—C13	118.83 (16)	O4—C50—H50A	110.0
C14—C15—C16	117.95 (17)	C49—C50—H50A	110.0
C14—C15—C23	123.50 (16)	O4—C50—H50B	110.0
C16—C15—C23	118.40 (16)	C49—C50—H50B	110.0
C17—C16—C15	122.51 (18)	H50A—C50—H50B	108.4
C17—C16—H16	118.7	C25—O4—C50	113.27 (14)
C15—C16—H16	118.7	C14—O5—C51	113.19 (14)
C16—C17—C18	117.38 (18)	O5—C51—C52	110.60 (15)
C16—C17—C19	122.69 (18)	O5—C51—H51A	109.5
C18—C17—C19	119.90 (18)	C52—C51—H51A	109.5
C13—C18—C17	122.43 (18)	O5—C51—H51B	109.5
C13—C18—H18	118.8	C52—C51—H51B	109.5
C17—C18—H18	118.8	H51A—C51—H51B	108.1
C22—C19—C17	112.65 (18)	N1—C52—C56	122.70 (18)
C22—C19—C20	108.6 (2)	N1—C52—C51	115.58 (17)
C17—C19—C20	108.70 (18)	C56—C52—C51	121.62 (18)
C22—C19—C21	108.4 (2)	C53—N1—C52	117.53 (18)
C17—C19—C21	109.11 (18)	N1—C53—C54	123.8 (2)
C20—C19—C21	109.3 (2)	N1—C53—H53	118.1
C19—C20—H20A	109.5	C54—C53—H53	118.1
C19—C20—H20B	109.5	C53—C54—C55	118.5 (2)
H20A—C20—H20B	109.5	C53—C54—H54	120.8
C19—C20—H20C	109.5	C55—C54—H54	120.8
H20A—C20—H20C	109.5	C54—C55—C56	118.9 (2)
H20B—C20—H20C	109.5	C54—C55—H55	120.6
C19—C21—H21A	109.5	C56—C55—H55	120.6
C19—C21—H21B	109.5	C55—C56—C52	118.64 (19)
H21A—C21—H21B	109.5	C55—C56—H56	120.7
C19—C21—H21C	109.5	C52—C56—H56	120.7
H21A—C21—H21C	109.5	C36—O6—C57	113.85 (14)
H21B—C21—H21C	109.5	O6—C57—C58	110.95 (15)
C19—C22—H22A	109.5	O6—C57—H57A	109.4
C19—C22—H22B	109.5	C58—C57—H57A	109.5
H22A—C22—H22B	109.5	O6—C57—H57B	109.5
C19—C22—H22C	109.5	C58—C57—H57B	109.4
H22A—C22—H22C	109.5	H57A—C57—H57B	108.0
H22B—C22—H22C	109.5	N2—C58—C62	122.41 (19)
C24—C23—C15	108.56 (15)	N2—C58—C57	116.37 (17)
C24—C23—H23A	110.0	C62—C58—C57	121.02 (18)
C15—C23—H23A	110.0	C59—N2—C58	117.68 (19)
C24—C23—H23B	110.0	N2—C59—C60	123.4 (2)
C15—C23—H23B	110.0	N2—C59—H59	118.3
H23A—C23—H23B	108.4	C60—C59—H59	118.3
C29—C24—C25	117.87 (17)	C61—C60—C59	118.5 (2)
C29—C24—C23	120.68 (16)	C61—C60—H60	120.8
C25—C24—C23	121.14 (16)	C59—C60—H60	120.8
O4—C25—C24	118.04 (16)	C60—C61—C62	119.0 (2)
O4—C25—C26	119.89 (16)	C60—C61—H61	120.5
C24—C25—C26	122.06 (16)	C62—C61—H61	120.5
C25—C26—C27	117.68 (16)	C61—C62—C58	118.9 (2)
C25—C26—C34	121.30 (16)	C61—C62—H62	120.6
C27—C26—C34	120.99 (17)	C58—C62—H62	120.6
C28—C27—C26	122.05 (17)	C72—O7—H7O7	109.5
C28—C27—H27	119.0	O7—C72—H72A	109.5
C26—C27—H27	119.0	O7—C72—H72B	109.5
C27—C28—C29	117.82 (17)	H72A—C72—H72B	109.5
C27—C28—C30	122.92 (17)	O7—C72—H72C	109.5
C29—C28—C30	119.25 (17)	H72A—C72—H72C	109.5
C24—C29—C28	122.10 (17)	H72B—C72—H72C	109.5
C24—C29—H29	118.9	C73—O8—H8O8	109.5
C28—C29—H29	118.9	O8—C73—H73A	109.5
C33—C30—C28	112.45 (17)	O8—C73—H73B	109.5
C33—C30—C32	108.7 (2)	H73A—C73—H73B	109.5
C28—C30—C32	109.79 (17)	O8—C73—H73C	109.5
C33—C30—C31	108.6 (2)	H73A—C73—H73C	109.5
C28—C30—C31	107.87 (17)	H73B—C73—H73C	109.5
			
C68—C63—C64—F1	179.13 (19)	C29—C24—C25—C26	−7.3 (3)
I1—C63—C64—F1	2.5 (3)	C23—C24—C25—C26	166.34 (17)
C68—C63—C64—C65	1.4 (3)	O4—C25—C26—C27	−174.81 (16)
I1—C63—C64—C65	−175.26 (17)	C24—C25—C26—C27	6.3 (3)
F1—C64—C65—F2	0.0 (3)	O4—C25—C26—C34	7.4 (3)
C63—C64—C65—F2	177.8 (2)	C24—C25—C26—C34	−171.47 (17)
F1—C64—C65—C66	−177.5 (2)	C25—C26—C27—C28	−0.8 (3)
C63—C64—C65—C66	0.3 (3)	C34—C26—C27—C28	176.97 (17)
F2—C65—C66—C67	−179.1 (2)	C26—C27—C28—C29	−3.4 (3)
C64—C65—C66—C67	−1.6 (3)	C26—C27—C28—C30	176.19 (18)
F2—C65—C66—I2	−3.3 (3)	C25—C24—C29—C28	2.8 (3)
C64—C65—C66—I2	174.18 (17)	C23—C24—C29—C28	−170.86 (17)
C65—C66—C67—F3	179.5 (2)	C27—C28—C29—C24	2.4 (3)
I2—C66—C67—F3	3.7 (3)	C30—C28—C29—C24	−177.21 (17)
C65—C66—C67—C68	1.3 (3)	C27—C28—C30—C33	3.2 (3)
I2—C66—C67—C68	−174.51 (16)	C29—C28—C30—C33	−177.3 (2)
F3—C67—C68—F4	0.0 (3)	C27—C28—C30—C32	124.4 (2)
C66—C67—C68—F4	178.21 (19)	C29—C28—C30—C32	−56.1 (2)
F3—C67—C68—C63	−177.80 (19)	C27—C28—C30—C31	−116.6 (2)
C66—C67—C68—C63	0.4 (3)	C29—C28—C30—C31	62.9 (2)
C64—C63—C68—F4	−179.50 (19)	C25—C26—C34—C35	93.1 (2)
I1—C63—C68—F4	−2.9 (3)	C27—C26—C34—C35	−84.6 (2)
C64—C63—C68—C67	−1.8 (3)	C26—C34—C35—C40	77.5 (2)
I1—C63—C68—C67	174.88 (16)	C26—C34—C35—C36	−101.9 (2)
C71^i^—C69—C70—F5	179.9 (2)	C40—C35—C36—C37	−3.9 (3)
I3—C69—C70—F5	−0.3 (3)	C34—C35—C36—C37	175.61 (17)
C71^i^—C69—C70—C71	−0.9 (4)	C40—C35—C36—O6	177.93 (16)
I3—C69—C70—C71	178.94 (17)	C34—C35—C36—O6	−2.6 (3)
F5—C70—C71—F6	0.0 (3)	O6—C36—C37—C38	−175.90 (16)
C69—C70—C71—F6	−179.2 (2)	C35—C36—C37—C38	5.9 (3)
F5—C70—C71—C69^i^	−179.9 (2)	O6—C36—C37—C1	9.7 (3)
C69—C70—C71—C69^i^	0.9 (4)	C35—C36—C37—C1	−168.53 (17)
C37—C1—C2—C3	−92.9 (2)	C2—C1—C37—C36	99.8 (2)
C37—C1—C2—C7	80.3 (2)	C2—C1—C37—C38	−74.7 (2)
C7—C2—C3—O1	177.01 (16)	C36—C37—C38—C39	−3.7 (3)
C1—C2—C3—O1	−9.7 (3)	C1—C37—C38—C39	171.06 (17)
C7—C2—C3—C4	−4.6 (3)	C37—C38—C39—C40	−0.5 (3)
C1—C2—C3—C4	168.65 (17)	C37—C38—C39—C41	−179.11 (18)
O1—C3—C4—C5	−177.88 (16)	C36—C35—C40—C39	−0.6 (3)
C2—C3—C4—C5	3.8 (3)	C34—C35—C40—C39	179.94 (17)
O1—C3—C4—C12	5.7 (3)	C38—C39—C40—C35	2.7 (3)
C2—C3—C4—C12	−172.64 (17)	C41—C39—C40—C35	−178.69 (18)
C3—C4—C5—C6	−0.7 (3)	C38—C39—C41—C44	−129.4 (2)
C12—C4—C5—C6	175.76 (17)	C40—C39—C41—C44	52.0 (2)
C4—C5—C6—C7	−1.3 (3)	C38—C39—C41—C42	−10.1 (3)
C4—C5—C6—C8	−178.73 (18)	C40—C39—C41—C42	171.41 (19)
C5—C6—C7—C2	0.4 (3)	C38—C39—C41—C43	111.1 (2)
C8—C6—C7—C2	177.78 (18)	C40—C39—C41—C43	−67.4 (2)
C3—C2—C7—C6	2.5 (3)	C2—C3—O1—C45	−94.2 (2)
C1—C2—C7—C6	−170.87 (17)	C4—C3—O1—C45	87.4 (2)
C7—C6—C8—C9	127.5 (2)	C3—O1—C45—C46	157.52 (16)
C5—C6—C8—C9	−55.3 (3)	O1—C45—C46—O2	54.9 (2)
C7—C6—C8—C11	−112.4 (2)	C45—C46—O2—C47	179.18 (16)
C5—C6—C8—C11	64.9 (3)	C46—O2—C47—C48	167.37 (16)
C7—C6—C8—C10	8.1 (3)	O2—C47—C48—O3	−62.3 (2)
C5—C6—C8—C10	−174.66 (19)	C47—C48—O3—C49	173.38 (16)
C5—C4—C12—C13	−82.9 (2)	C48—O3—C49—C50	−174.10 (15)
C3—C4—C12—C13	93.5 (2)	O3—C49—C50—O4	53.3 (2)
C4—C12—C13—C18	77.2 (2)	C24—C25—O4—C50	−96.29 (19)
C4—C12—C13—C14	−100.6 (2)	C26—C25—O4—C50	84.8 (2)
C18—C13—C14—C15	−4.9 (3)	C49—C50—O4—C25	154.11 (15)
C12—C13—C14—C15	172.94 (17)	C15—C14—O5—C51	99.66 (19)
C18—C13—C14—O5	176.02 (16)	C13—C14—O5—C51	−81.2 (2)
C12—C13—C14—O5	−6.2 (3)	C14—O5—C51—C52	−80.68 (19)
O5—C14—C15—C16	−175.64 (16)	O5—C51—C52—N1	−84.9 (2)
C13—C14—C15—C16	5.3 (3)	O5—C51—C52—C56	91.6 (2)
O5—C14—C15—C23	8.8 (3)	C56—C52—N1—C53	−0.6 (3)
C13—C14—C15—C23	−170.32 (17)	C51—C52—N1—C53	175.90 (18)
C14—C15—C16—C17	−2.0 (3)	C52—N1—C53—C54	−0.3 (3)
C23—C15—C16—C17	173.79 (18)	N1—C53—C54—C55	0.9 (4)
C15—C16—C17—C18	−1.5 (3)	C53—C54—C55—C56	−0.7 (3)
C15—C16—C17—C19	−179.23 (19)	C54—C55—C56—C52	−0.2 (3)
C14—C13—C18—C17	1.2 (3)	N1—C52—C56—C55	0.9 (3)
C12—C13—C18—C17	−176.70 (18)	C51—C52—C56—C55	−175.48 (19)
C16—C17—C18—C13	1.9 (3)	C37—C36—O6—C57	80.4 (2)
C19—C17—C18—C13	179.72 (19)	C35—C36—O6—C57	−101.37 (19)
C16—C17—C19—C22	−4.8 (3)	C36—O6—C57—C58	70.3 (2)
C18—C17—C19—C22	177.5 (2)	O6—C57—C58—N2	−89.6 (2)
C16—C17—C19—C20	115.6 (2)	O6—C57—C58—C62	85.5 (2)
C18—C17—C19—C20	−62.1 (3)	C62—C58—N2—C59	−3.9 (3)
C16—C17—C19—C21	−125.3 (2)	C57—C58—N2—C59	171.15 (19)
C18—C17—C19—C21	57.0 (3)	C58—N2—C59—C60	−0.2 (3)
C14—C15—C23—C24	102.5 (2)	N2—C59—C60—C61	3.8 (4)
C16—C15—C23—C24	−73.0 (2)	C59—C60—C61—C62	−3.5 (3)
C15—C23—C24—C29	80.7 (2)	C60—C61—C62—C58	−0.3 (3)
C15—C23—C24—C25	−92.8 (2)	N2—C58—C62—C61	4.1 (3)
C29—C24—C25—O4	173.84 (16)	C57—C58—C62—C61	−170.66 (18)
C23—C24—C25—O4	−12.5 (2)		

Symmetry code: (i) −*x*+1, −*y*, −*z*+1.

### Hydrogen-bond geometry (Å, º)

**Table:**

*D*—H···*A*	*D*—H	H···*A*	*D*···*A*	*D*—H···*A*
C63—I1···O7		2.75 (1)		179 (1)
C69—I3···I4		3.38 (1)		175 (1)
C66—I2···F6^ii^		3.21 (1)		149 (1)
O7—H7*O*7···N2	0.84	2.00	2.809 (3)	161

Symmetry code: (ii) *x*, *y*+1, *z*−1.
